# Assessing costs of carrying geolocators using feather corticosterone in two species of aerial insectivore

**DOI:** 10.1098/rsos.150004

**Published:** 2015-05-06

**Authors:** Graham D. Fairhurst, Lisha L. Berzins, David W. Bradley, Andrew J. Laughlin, Andrea Romano, Maria Romano, Chiara Scandolara, Roberto Ambrosini, Russell D. Dawson, Peter O. Dunn, Keith A. Hobson, Felix Liechti, Tracy A. Marchant, D. Ryan Norris, Diego Rubolini, Nicola Saino, Caz M. Taylor, Linda A. Whittingham, Robert G. Clark

**Affiliations:** 1 Department of Biology, University of Saskatchewan, 112 Science Place, Saskatoon, Saskatchewan, Canada S7N 5E2; 2 Environment Canada, 115 Perimeter Road, Saskatoon, Saskatchewan, Canada S7N 0X4; 3 Ecosystem Science and Management, University of Northern British Columbia, 3333 University Way, Prince George, British Columbia, Canada V2N 4Z9; 4 Department of Integrative Biology, University of Guelph, 50 Stone Road East, Guelph, Ontario, Canada N1G 1E4; 5 Bird Studies Canada, 115 Front Street, Port Rowan, Ontario, Canada N0E 1M0; 6 Department of Ecology and Evolutionary Biology, Tulane University, New Orleans, LA 70118, USA; 7 Department of Biosciences, University of Milan, via Celoria 26, Milan 20133, Italy; 8 Swiss Ornithological Institute, Seerose 1, Sempach 6204, Switzerland; 9 Department of Biotechnology and Biosciences, University of Milano Bicocca, Piazza della Scienza 2, Milan 20126, Italy; 10 Department of Biological Sciences, University of Wisconsin—Milwaukee, 3209 North Maryland Avenue, Milwaukee, WI 53201-0413, USA; 11 Environment Canada, 11 Innovation Boulevard, Saskatoon, Saskatchewan, Canada S7N 3H5

**Keywords:** Energetic Expenditure Hypothesis, feather corticosterone, hormone biomarkers, light-level geolocators, migration physiology, swallows

## Abstract

Despite benefits of using light-sensitive geolocators to track animal movements and describe patterns of migratory connectivity, concerns have been raised about negative effects of these devices, particularly in small species of aerial insectivore. Geolocators may act as handicaps that increase energetic expenditure, which could explain reported effects of geolocators on survival. We tested this ‘Energetic Expenditure Hypothesis’ in 12 populations of tree swallows (*Tachycineta bicolor*) and barn swallows (*Hirundo rustica*) from North America and Europe, using measurements of corticosterone from feathers (CORT_f_) grown after deployment of geolocators as a measure of physiology relevant to energetics. Contrary to predictions, neither among- (both species) nor within-individual (tree swallows only) levels of CORT_f_ differed with respect to instrumentation. Thus, to the extent that CORT_f_ reflects energetic expenditure, geolocators apparently were not a strong handicap for birds that returned post-deployment. While this physiological evidence suggests that information about migration obtained from returning geolocator-equipped swallows is unbiased with regard to levels of stress, we cannot discount the possibility that corticosterone played a role in reported effects of geolocators on survival in birds, and suggest that future studies relate corticosterone to antecedent factors, such as reproductive history, and to downstream fitness costs.

## 2. Introduction

Understanding the ecological and population processes affecting migratory birds requires knowledge of habitat use and individual movements throughout the annual cycle [1–3]. Recent insights have been facilitated by advances in techniques for tracking animal movements and describing patterns of migratory connectivity [4–7]. The use of light-sensitive geolocators has become especially popular because the devices now weigh less than 1 g and, therefore, can be used on many species of small-bodied migratory passerines [8]. Indeed, the recent rapid increase in research using geolocators has revealed previously unknown information about breeding areas [9], migratory routes and stopover areas [10,11], non-breeding areas [12–14] and migratory connectivity [9,12,15] for a variety of small bird species [8].

Despite obvious benefits of using geolocators to track migration, concerns have been raised about negative effects of these devices and the potential biases in data derived from them [16–19]. A recent meta-analysis provided evidence that geolocators can reduce survival, particularly for aerial foragers and migratory species [17]. Effects of geolocators on flight mechanics can help explain these findings and include increased wing loading and drag owing to altered aerodynamic profiles [20,21]. To compensate for these effects, individuals carrying geolocators would be expected to increase energetic expenditure [16,22,23]. This added workload could be particularly taxing during migration, which is a period of high energetic demand [22,24] and high mortality [25]. Thus, geolocators have the potential to detrimentally influence the energetic balance of migrants.

Although this ‘Energetic Expenditure Hypothesis’ may provide a reasonable mechanism for reported effects of geolocators, testing it requires measuring the energetics of free-living birds following deployment. Unlike other tracking technologies [26], current geolocators suitable for use with small birds (i.e. devices<1.0 g) cannot collect any biotelemetry data other than location. Moreover, most small migrant passerines cannot be recaptured until they return to the breeding grounds. These issues make it difficult to assess differences in *en route* physiology of individuals with and without geolocators, which is critical for establishing or refuting a physiological link between geolocators and variation in performance measures affecting fitness.

The hormone corticosterone (CORT) may be a useful proxy for measuring the effect of geolocators on the energetics of migratory birds. CORT is a metabolic hormone well known for its role in energy management [27,28], and CORT levels rise in response to increased energetic demands and facilitate the conversion (and thus depletion) of energy stores into usable forms [29–33]. In migratory passerines, CORT levels are elevated seasonally to meet the physiological demands of migration, but birds still respond to stressors during this period [33–35] and during winter [36,37]. Thus, if instrumentation with a geolocator acts as a handicap that unpredictably increases energetic demands, CORT levels could rise to a point where costs, such as increased catabolism of energy stores, degradation of muscle and immunosuppression occur [28,38]. Short-term effects of geolocators and other tracking devices on CORT physiology are either ephemeral (e.g. [39,40]) or not detectable (e.g. [41]), but studies of seabirds show that baseline and handling-induced CORT levels are significantly elevated the year following geolocator deployment [23,42]. All of these previous studies measured CORT during the breeding season (or in captivity) so we lack any assessment of the effects of geolocators on energetics outside of this period in wild populations.

Feathers may provide a retrospective ‘remote sensing’ of avian energetics because they contain a record of CORT during the period of feather growth [43]. The CORT in feathers (CORT_f_) has been shown experimentally to reflect levels of plasma CORT [44,45] and a variety of stressors [46–50] during feather growth. For birds that moult after the deployment of geolocators, CORT_f_ could quantify a physiologically relevant proxy of energetic costs arising from instrumentation. In migratory passerines, assessing energetics during the pre-basic moult, which itself is energetically expensive [51,52], may be particularly pertinent because this moult is preceded by physiologically demanding activities (e.g. breeding, migration or both), the energetic costs of which could carry over into the moulting period. Therefore, CORT_f_ may reflect cumulative energetic costs that could be more pronounced in birds carrying geolocators.

Using CORT_f_ as a measure of physiological response to geolocators, we tested two predictions of the Energetic Expenditure Hypothesis: (i) compared with returning adults without geolocators from the same population (controls), individuals returning with geolocators (geolocator birds) should have higher levels of CORT_f_, reflecting their increased energetic expenditure; and (ii) within individuals carrying a geolocator, post-deployment levels of CORT_f_ should be higher than pre-deployment levels. As a group, small aerial insectivores should be particularly susceptible to the effects of geolocators [17], making them appropriate models for this type of study. Thus, we tested our predictions in four populations of tree swallows (*Tachycineta bicolor*) and eight populations of barn swallows (*Hirundo rustica*) from North America and Europe. By studying how physiology of migratory passerines varies in response to geolocators, this study also provides data useful for resolving potential ethical and scientific issues facing researchers tracking small birds over long distances.

## 3. Material and methods

### 3.1 Fieldwork

Complete details of field methods, including geolocator instrumentation, for the birds in our study have been presented elsewhere (tree swallows [11,18]; barn swallows [19,53]). For tree swallows, fieldwork was conducted during May–July of 2011–2013 at three breeding sites in Canada (Prince George, British Columbia: 53°50′ N, 122°57′ W; St Denis National Wildlife Area, Saskatchewan: 52°13′ N, 106°04′ W; Long Point, Ontario: 42°39′ N, 80°26′ W) and one in the USA (Saukville, Wisconsin: 43°24′ N, 88°0′ W). Adults were captured at their nest-boxes during the brood-rearing period and individuals were banded, sexed, measured and dorsal contour feathers were collected from the upper back using forceps and stored in paper envelopes until subsequent CORT analyses. Geolocators (0.67 g; Lotek Wireless model MK12-S in 2011, MK5-S in 2012) were attached using a modified leg-loop backpack harness [10], composed of 1 mm diameter solid ethylenepropylene-diene rubber tubing, that had a combined mass of less than or equal to 1.0 g (less than 5% of body mass). The geolocator, which sat just anterior to the tail, was secured to the contour feathers on the bird's back using a small amount of cyanoacrylate adhesive and did not directly impede movement of the wings. Different adult tree swallows were marked with geolocators in 2011 and 2012.

For barn swallows, fieldwork in North America was conducted during May–July of 2012 and 2013 at two breeding sites in Canada (Prince Albert National Park, Saskatchewan: 53°42′ N, 106°3′ W; near Sackville, New Brunswick: 45°58′ N, 64°13′ W) and three in the USA (Auburn, Alabama: 32°33′ N, 85°21′ W; Greenville, Mississippi: 33°17′ N, 91°2′ W; Seattle, Washington: 47°39′ N, 122°21′ W). Fieldwork in Europe took place during April–July of 2010–2012 at one breeding site in southern Switzerland (Magadino: 46°09′ N, 8°55′ W) and two in northern Italy (Piedmont: 45°33′ N, 8°44′ E; Lombardy: 45°19′ N, 9°40′ E). Adults were captured with mist-nets, individually marked with coloured leg bands, sexed, measured and the fourth outermost tail feather (R4) was plucked and stored for CORT analysis. For North American breeding sites, geolocators (0.7 g; Migrate Technology model Intigeo-P55B1–7) were deployed at this time and were attached using a leg-loop harness composed of elastic cord (Stretch Magic, Pepperell, MA, USA). The combined mass was less than 0.8 g (approx. 4.5% of body mass). For European breeding sites, adults were recaptured at the end of the breeding season and geolocators (Swiss Ornithological Institute model SOI-GDL2.10 in 2010, SOI-GDL2.11 in 2011) were deployed. Geolocators (2010: approx. 0.77 g; 2011: approx. 0.68 g) were attached using a leg-loop harness composed of an elastic silicone rubber tubing, and the combined mass was less than 0.8 g (less than 4% of body mass).

### 3.2 Nomenclature and sample sizes of feathers

Feathers from geolocator birds were either grown the autumn before (pre-deployment) or after (post-deployment) deployment. The moulting of tree swallow back feathers occurs from mid-July to early November, corresponding to the beginning of autumn migration for the majority of individuals, and is probably completed within North America ([11,54,55] and references therein). Barn swallow tail feathers are moulted at the end of autumn migration on wintering grounds in Africa and South America ([55–57] and references therein).

As not all geolocator birds returned the year following deployment, for among-individual analyses of tree swallows we had four categories of feathers that comprised treatment groups: (i) feathers from controls reflecting the general population of returning individuals, (ii) post-deployment feathers from geolocator birds that returned the subsequent year, (iii) pre-deployment feathers from returning geolocator birds, and (iv) pre-deployment feathers from geolocator birds that did not return. Groups (iii) and (iv) are analogous to controls; analysing them separately enabled us to determine if differences in CORT physiology existed in these treatments prior to deployment (see Statistical analyses section). We had feathers from 40 tree swallows recaptured the year subsequent to their original sampling (control: *n*=12 birds; geolocator: *n*=28 birds). We did not have any pre-deployment feathers for barn swallows, and thus only had feathers in two treatment groups: (i) feathers from controls reflecting the general population of returning individuals and (ii) post-deployment feathers from geolocator birds that returned the subsequent year. Sample sizes for each treatment described above are presented in table 1.

**Table 1.** Sample sizes of feathers from each species, population, year and sex (male/female) in each treatment group. (See text for explanation of treatments.)

population	year	control	pre-deployment from non-returning geolocator birds	pre-deployment from returning geolocator birds	post-deployment from geolocator birds	population total
tree swallows						
Long Point, ON	2011	0	0	2/6	0	8
	2012	0/1	0	0	6/7	14
	2013	3/5	0	0	2/2	12
	total	3/6	0	2/6	8/9	34
St Denis, SK	2011	0	0	0	0	0
	2012	4/0	0	0	8/2	14
	2013	0	0	0	0	0
	total	4/0	0	0	8/2	14
Prince George, BC	2011	0/2	0	0/2	0	4
	2012	5/7	5/11	5/4	0/1	38
	2013	5/5	0	0	5/5	20
	total	10/14	5/11	5/6	5/6	62
Saukville, WI	2011	0/0	13/16	9/1	0	39
	2012	2/8	0	0	9/1	20
	total	2/8	13/16	9/1	9/1	59
barn swallows						
Auburn, AL	2013	1/8/3^a^	0	0	2/0	14
Greenville, MS	2013	7/9	0	0	0/1	17
Seattle, WA	2013	6/6	0	0	1/1	14
Sackville, NB	2013	6/9	0	0	0/2	17
Prince Albert NP, SK	2013	7/6	0	0	1/1	15
Lombardy, IT	2012	6/3	0	0	12/4	25
	2013	2/0	0	0	2/0	4
	total	8/3	0	0	14/4	29
Piedmont, IT	2012	11/3	0	0	11/3	28
	2013	8/0	0	0	8/0	16
	total	19/3	0	0	19/3	44
Magadino, CH	2012	13/3	0	0	13/3	32

^a^unknown sex.

For tree swallows, geolocator birds were randomly selected from previously banded adults. Control birds were selected as the next banded adult captured, which was generally the same day or shortly after deployment of a geolocator, so control and geolocator birds were well matched in their timing of breeding. For barn swallows, in 2010, birds were assigned alternately to control or geolocator groups within each colony of each breeding site. In 2011, this procedure was maintained at the Magadino and Piedmont breeding sites, but at the Lombardy site different breeding colonies were assigned to different treatment groups for practical reasons. Regardless, in both years and at all sites, birds in the two treatment groups were well matched in their timing of breeding.

### 3.3 Analysis of corticosterone from feathers

Analyses of CORT_f_ were conducted as in previous studies of tree swallows [44,58]. We first processed feathers by removing the calamus, weighing and measuring the length of the remaining portion of feather, placing each sample into a separate glass vial, and cutting the samples into small pieces with scissors. We then added 10 ml of HPLC-grade methanol (VWR International, Mississauga, Ontario, Canada) to each sample, sonicated all samples at room temperature for 30 min, followed by incubation at 50°C overnight in a water bath. A vacuum filtration system consisting of a plug of polyester wool in a glass filtration funnel was used to separate the methanol extract from the feather material. The original sample vial, remnant feather pieces and filtration material were washed twice with approximately 2.5 ml of additional methanol that was then added to the original methanol extract. Methanol extracts were placed in a 50°C water bath and subsequently evaporated in a fume hood. Samples were extracted in six batches. Recovery efficiency of the methanol extraction was assessed by including feather samples spiked with approximately 5000 CPM of ^3^H-labelled CORT, and an average of 93.4% (s.d.=6.1) of the radioactivity was recoverable in the reconstituted samples. Samples were adjusted for recoveries. Extract residues were reconstituted in a small volume of phosphate buffer (0.05 mol l^−1^, pH 7.6) and analysed by radioimmunoassay in duplicate following [59]. Serial dilutions of sample extracts of both species were parallel to the standard curve, indicating no interference with the antibody. All samples were run blind with regard to individual identity. Samples from all populations except Saukville, WI, were randomly distributed throughout five assays, and the average intra-assay variability, computed using three aliquots per assay of the same standard, was 8.8% (s.d.=5.4), inter-assay variability was 9.1%, and all samples were above the limit of detection (ED_80_; average±s.d.: 16.08±2.42 pg 100 μl^−1^). Saukville samples were obtained 1 year later and randomized throughout a single assay run with a different internal standard but same antiserum as all previous samples. Our statistical analyses do not compare CORT_f_ values among populations (we intentionally control for this using population as a random effect; see Statistical analyses section) and are instead tested for differences among treatments within sites. This single assay had an intra-assay variability of 5.7% (i.e. was internally valid) and all samples were above its limit of detection (ED_80_) of 12.99 pg 100 μl^−1^ (similar to the other assays). CORT_f_ values were standardized by feather length (i.e. CORT mm^−1^) to best represent the time-dependent deposition of CORT [43,60,61].

### 3.4 Statistical analyses

CORT_f_ values were log-transformed to improve normality. We used mixed models to analyse the effect of geolocators on CORT_f_, using Proc Mixed in SAS v. 9.2 (SAS Institute, Cary, NC, USA), including population and year as random effects to account for clustered data and annual effects. Owing to the unbalanced sample sizes among treatment, sex, year and population, all mixed models used the Kenward-Rogers method for approximating degrees of freedom. Non-significant interaction terms (*p*>0.05) were eliminated from models. Because different types of feathers were used for tree swallows and barn swallows, we analysed each species separately.

For both species, we first examined the variation in CORT_f_ among treatments. These models started with fixed effects of treatment (for definitions see Nomenclature and sample sizes section), minimum age (youngest reliably estimable age) and sex, and included a treatment×sex interaction. Only known-sex birds were used in analyses that included sex. Second, for tree swallows alone, we addressed within-individual effects of instrumentation with a geolocator using the 40 individuals sampled in two consecutive years. Each bird was used as its own control by subtracting pre-deployment (year 1) values from post-deployment (year 2) values. This created a single variable that reflected the within-individual change in CORT physiology from one year to the next. We compared this variable between geolocator and control birds in a model that also included the fixed effects of minimum age and sex, and a treatment×sex interaction.

## 4. Results

**Figure 1.**
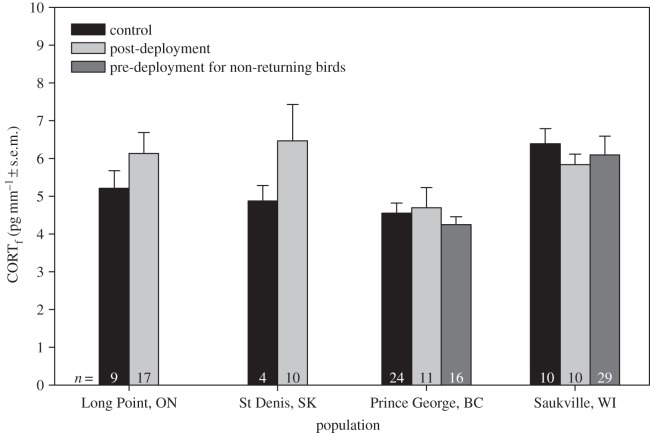
Levels of corticosterone in feathers (CORT_f_) from tree swallows instrumented with a geolocator compared to non-instrumented (control) birds. All feathers were grown post-breeding, and pre-deployment feathers were grown the year prior to deployment of geolocators. See text for complete descriptions of treatments. Note that some populations contain multiple years of data (table 1).

**Figure 2.**
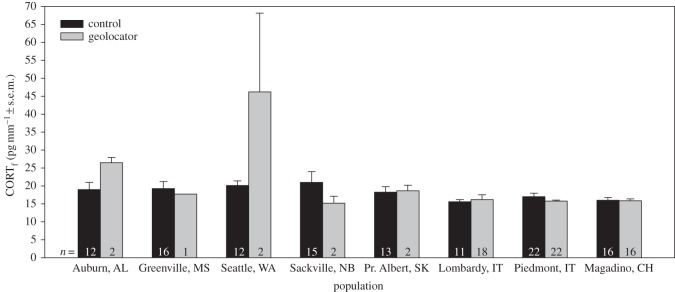
Levels of corticosterone in feathers (CORT_f_) from barn swallows instrumented with a geolocator compared to controls. All feathers were grown post-breeding (i.e. after deployment of geolocators). Note that some populations contain multiple years of data (table 1).

In tree swallows, there was no interaction between treatment and sex on CORT_f_ (*F*_3,152_=0.22, *p*=0.88), so the interaction term was removed from the model. The final model revealed no effect of geolocators on CORT_f_ (*F*_3,149_=0.69, *p*=0.56; figure 1), and no effect of sex (*F*_1,154_=1.14, *p*=0.29) or minimum age (*F*_8,153_=0.45, *p*=0.89). In barn swallows, there was no interaction between treatment and sex on CORT_f_ (*F*_1,169_=0.07, *p*=0.79), so this term was also removed from the model. The final model showed no effect of geolocators on CORT_f_ (*F*_1,171_=0.47, *p*=0.49; figure 2), and no effect of sex (*F*_1,170_=2.45, *p*=0.12) or age (*F*_5,170_=0.47, *p*=0.80).

**Figure 3.**
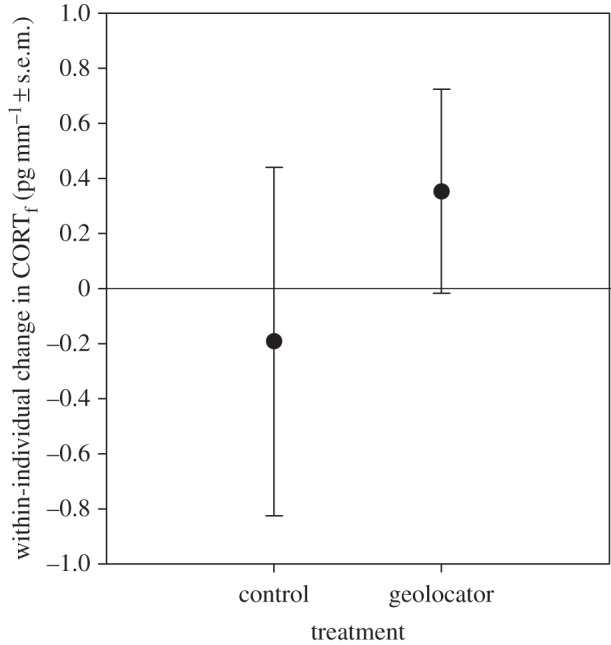
Within-individual change in levels of corticosterone from feathers (CORT_f_) of tree swallows in two consecutive years. For control birds (*n*=12), this is the change in levels from year 1 to year 2; for geolocator birds (*n*=28), this reflects the change from pre-deployment (year 1) to post-deployment (year 2) levels.

When we considered the 40 cases where tree swallows were sampled in two consecutive years, we found that within-individual changes in CORT_f_ were not related to the interaction of treatment with sex (*F*_1,30_=1.80, *p*=0.19) so this term was removed from the model. The final model revealed no effect of geolocators on within-individual changes in CORT_f_ from one year to the next (*F*_1,31_=0.28, *p*=0.60; figure 3), and no effects of age (*F*_6,31_=0.27, *p*=0.95) or sex (*F*_1,31_=0.53, *p*=0.47).

## 5. Discussion

We tested the Energetic Expenditure Hypothesis that geolocators attached to aerial insectivores produce a handicap that increases energetic demand. We predicted that if there was a pervasive effect of geolocators it would be reflected in levels of CORT from feathers grown prior to or early-on during post-breeding migration (tree swallows), or at the end of migration (barn swallows). We also expected that our broad geographical and temporal approach of analysing 3 years of CORT_f_ data from 12 populations of two species of aerial insectivore on two continents would provide the power to detect an effect of geolocators if one existed. However, our results based on both among-individual (both species) and within-individual (tree swallows only) analyses show that there was no effect of geolocators on levels of CORT_f_. The lack of effect in barn swallows is particularly revealing, considering that they carried the geolocator for considerably longer before moulting than did tree swallows. Thus, to the extent that CORT_f_ reflects energetic expenditure, our findings suggest that geolocators apparently did not act as a strong handicap for birds that returned post-deployment. We further speculate that this provides physiological evidence that data about locations and timing of migration obtained from returning geolocator birds (e.g. [11]) may not be biased with regard to levels of stress, although this should be tested directly.

Our findings do not rule out, however, an effect of geolocators on CORT physiology, nor do they necessarily discount CORT as a potential mediator of the effects of geolocators on survival. If non-returning geolocator birds had CORT physiology operating in homeostatic overload (*sensu* [62]) for extended periods, then they could have experienced costs including reduced condition, increased susceptibility to disease or death (for reviews see [28,38]). Sub-lethal effects of CORT could have reduced the ability of these birds to acquire resources during stopovers or on the wintering grounds, or influenced their decision to not travel as far as controls, resulting in lower return rates to breeding grounds the subsequent year, which have been detected in several of our populations [18,19]. Moreover, factors operating prior to departure from the breeding grounds could have predisposed non-returning geolocator birds to potential negative effects arising from instrumentation. For example, reproductive effort can influence CORT physiology during and at the end of the breeding season [63,64] which, in turn, can have consequences for migration phenology [65,66]. If reproduction was particularly energetically demanding for non-returning geolocator birds (i.e. CORT levels were already near homeostatic overload), then geolocators could have further increased CORT levels and exacerbated costs. Although behavioural data indicate that control and geolocator tree swallows do not appear to differ immediately after instrumentation [18], physiological costs could have carried over into migration which would further increase energetic demands. The duration, speed and distance of the migratory journey, as well as habitat use during stopovers and on wintering grounds, can influence energetics, CORT physiology and return rates of birds [36,37,67–69]. Indeed, migration distance is believed to influence apparent survival rates of geolocator-marked birds [17], and CORT_f_ could possibly predict the pace of autumn and spring migration in tree swallows and barn swallows, respectively [65]. Thus, the ecophysiological context before, during and after migration is important for fully understanding how and when geolocators influence survival, the potential fitness consequences to survivors, and the extent to which CORT physiology is involved in these processes.

Regardless of the mechanism, individuals that were better able to manage their CORT physiology may have been better able to avoid costs [62] and thus survive. Measuring CORT from feathers grown post-deployment from non-returning geolocator birds is essential to substantiating this hypothesis but is not possible owing to difficulties recapturing swallows once they leave the breeding grounds. Thus, our ability to identify any obvious physiological differences between returning and non-returning geolocator birds is limited to comparing their pre-deployment CORT_f_ levels with controls, yet we found no differences among these three groups. Investigation of plasma CORT at the time of deployment should be a focus of future research. Although we lack evidence of physiological differences between returning and non-returning geolocator birds, it may be the case that only high-quality birds were instrumented to begin with, and this explains why CORT_f_ levels of returning geolocator birds did not differ from controls. This is a possibility for tree swallows because geolocators were deployed (albeit randomly) on previously banded birds that had already survived at least two migrations, but we can rule out this hypothesis for barn swallows because deployment of geolocators was completely randomized [19]. Nonetheless, it is important to note that, despite surviving and not having significantly higher levels of CORT_f_, returning geolocator birds may still have incurred a cost of instrumentation. Indeed, initial evidence in European populations of barn swallows suggests that geolocators impair subsequent reproduction [19]. It is unknown what role CORT plays in such effects, so future studies would benefit from determining whether body condition, health, or reproductive variables the spring following instrumentation vary with respect to CORT_f_ in returning geolocator birds.

Additional research is clearly needed to identify if physiological costs of instrumentation with geolocators exist and whether these influence survival, and the Energetic Expenditure Hypothesis provides testable predictions of such effects. To the extent that we can use CORT_f_ to infer variation in energetic expenditure, our results suggest that geolocators may not have imposed a handicap on returning swallows. Moreover, compared with birds that did not return and breed, returning birds did not have significantly different CORT_f_ in the year prior to instrumentation. Whether or not only the best-quality birds survived to be sampled and how CORT physiology may have contributed to this require future research. Longitudinal demographic studies such as ours are particularly informative for addressing how CORT_f_ relates to antecedent factors, such as reproductive history, and to downstream fitness costs. Validation studies are needed to determine if the levels of energetic exertion (or physiological stress) necessary to influence CORT_f_ are similar among species. As our understanding of migratory movements and stopover areas improve (e.g. [11]), it will become easier to sample geolocator and control birds throughout migration. Comparing physiological profiles of these birds at multiple stages throughout their journey will be essential to substantiating or refuting the Energetic Expenditure Hypothesis.

## Data accessibility

Data for this study have been deposited with Dryad ().
